# Melting of glacier ice enhanced by bursting air bubbles

**DOI:** 10.1038/s41561-023-01262-8

**Published:** 2023-09-07

**Authors:** Meagan E. Wengrove, Erin C. Pettit, Jonathan D. Nash, Rebecca H. Jackson, Eric D. Skyllingstad

**Affiliations:** 1 School of Civil and Construction Engineering, Oregon State University, Corvallis, OR USA; 2 College of Earth, Ocean, and Atmospheric Sciences, Oregon State University, Corvallis, OR USA; 3 Department of Marine and Coastal Sciences, Rutgers University, New Brunswick, NJ USA

**Keywords:** Physical oceanography, Cryospheric science, Physical oceanography

## Abstract

Feedbacks between ice melt, glacier flow and ocean circulation can rapidly accelerate ice loss at tidewater glaciers and alter projections of sea-level rise. At the core of these projections is a model for ice melt that neglects the fact that glacier ice contains pressurized bubbles of air due to its formation from compressed snow. Current model estimates can underpredict glacier melt at termini outside the region influenced by the subglacial discharge plume by a factor of 10–100 compared with observations. Here we use laboratory-scale experiments and theoretical arguments to show that the bursting of pressurized bubbles from glacier ice could be a source of this discrepancy. These bubbles eject air into the seawater, delivering additional buoyancy and impulses of turbulent kinetic energy to the boundary layer, accelerating ice melt. We show that real glacier ice melts 2.25 times faster than clear bubble-free ice when driven by natural convection in a laboratory setting. We extend these results to the geophysical scale to show how bubble dynamics contribute to ice melt from tidewater glaciers. Consequently, these results could increase the accuracy of modelled predictions of ice loss to better constrain sea-level rise projections globally.

## Abstract (web summary)

Laboratory experiments suggest that bursting bubbles enhance ice melt from tidewater glaciers, and consequently, glacier-ice structure needs to be accounted for in projections of ice loss and sea-level rise.

## Main

**Fig. 1:**
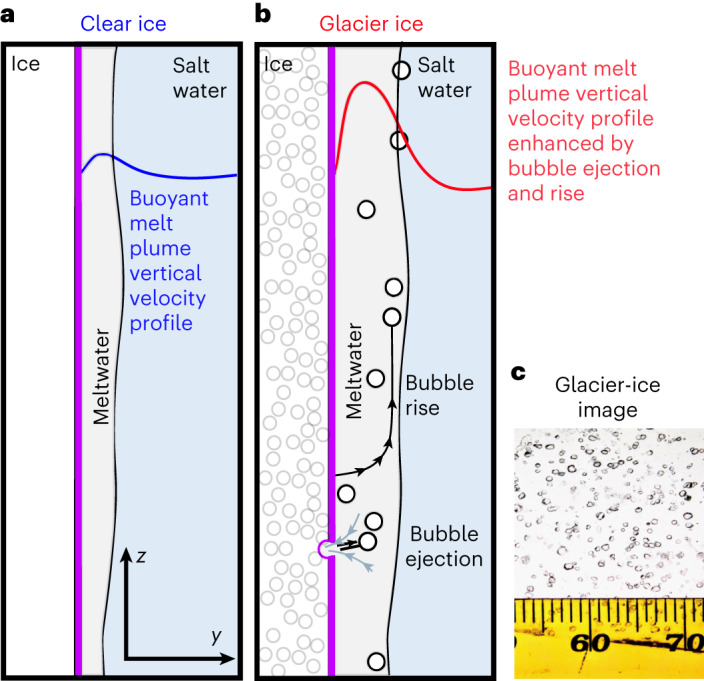
The influence of freshwater melt and bubble ejection on melt-plume signature for clear- (bubble-free) and glacier-ice melt. **a**, Clear-ice ambient melt plume. **b**, Glacier-ice ambient melt plume enhanced by the air injected into and rising with the boundary layer. **c**, Photograph of a 2-mm-thick thin-section slice of the Greenland glacier ice we used in these experiments. The image is 2 cm wide, the scale bar tick marks are 1 mm. Ice was collected as part of a study of past atmospheric gases^38,39^.

Tidewater glaciers are rapidly retreating, leading to ice loss in Greenland, the Antarctic Peninsula and other glacierized regions^1^. Projections for future sea-level rise due to the sustained mass loss of ice sheets have large uncertainties, in part because dynamic feedbacks where glaciers terminate into the ocean are poorly constrained^2–5^. In parallel, the prediction of ice-sheet melt has been shown to underestimate observed mass loss^4^, with recent reviews showing that the largest uncertainties are associated with ice–ocean feedbacks^3,4,6,7^. While predictions of submarine ice loss often use ocean temperature and subglacial discharge strength together as a proxy for melt^8–10^, recent studies suggest that substantial submarine melt occurs outside the region where the subglacial discharge plume is in contact with the ice, defined as ambient melt^11^. Observations at a tidewater glacier (Xeitl Sít’ in Tlingit, also known as LeConte Glacier, Alaska) show ambient melt to exceed prediction by the state-of-the-art parameterization by at least an order of magnitude^11,12^. Efforts to identify mechanisms for submarine melt across the tidewater glacier face have led to discoveries showing that enhanced near-boundary currents^11,13^, internal wave dynamics^14^ and variability in ice morphology^15^ may all be important to properly constrain tidewater glacier melt rates. Here we suggest that the microstructure of the glacier ice itself is an additional and important neglected factor, and that the most commonly used submarine melt parameterizations are missing important physics by assuming bubble-free ice. Specifically, we demonstrate how the bursting of pressurized bubbles from real glacier ice energizes the boundary layer at the vertical ice–ocean interface and enhances submarine melt (Fig. 1).

Glacier ice forms from compacted snow in the accumulation zone of a glacier or ice sheet. During compaction, air is trapped in pores between ice crystals and accounts for roughly 10% of the volume of newly formed ice^16^, typically with ~200 pores cm^−^^3^ (ref. ^17^; Fig. 1c). Once formed, glacier ice is impermeable to air, so air-filled pores (bubbles) become compressed and pressurized as the ice descends within a glacier or ice sheet^17–19^. The pressure within the bubbles depends on the depth (overburden pressure) along the path the ice takes through the glacier. Near the terminus, overburden pressure decreases, yet air in the bubbles retains excess pressure due to microstructural effects^17,20^ to as much as 20 bar^18^.

As glacier ice melts underwater, these pressurized bubbles explode and eject air into the seawater, creating audible pops (see Supplementary Video 1 and Supplementary Audio 1). As a result, tidewater glacier fjords are filled with acoustic noise^20^ that can be used as an indicator of ice melt^21–23^. Despite the well-known existence of pressurized bubbles in glacier ice, no studies have yet investigated their effect on near-boundary hydrodynamics. One early study^24^ used manufactured ice infused with CO_2_ at atmospheric pressure and concluded that bubbles had no measurable influence on melt rates. However, because the CO_2_ was at atmospheric pressure, we suspect the gas remained attached to the ice (rather than exploding away), minimizing the bubbles’ influence on the hydrodynamics. The results from that study may have contributed to the neglect of bubbles in all subsequent studies of ice melt and its parameterization in models. Since then, only one further study^25^ related melt rate to ice density (a proxy for total bubble volume), but did not investigate the underlying physics (see Supplementary Information for details of historical experiments). Recent laboratory^26–30^ and modelling experiments^31,32^ evaluate the turbulent statistics, temperature gradients and salinity gradients associated with melt in the adjacent seawater at vertical ice faces, but neglect the energy and buoyancy associated with bubble release.

Bubbles are known to affect mixing in many applications, from medicine and industrial processes to air–sea and seabed–water column interfaces^33–35^. However, until now, the impact of bubble bursts on glacier-ice melt dynamics has not been realized. Here we use laboratory-scale experiments to demonstrate the hydrodynamics of bubble ejection from natural glacier ice. Additionally, we introduce a physics-based energy analysis to quantify the influence of bubble ejection and rise as a function of terminus depth. These calculations suggest that bubbles may explain an important portion of the discrepancy between observed and predicted submarine tidewater glacier melt rates.

## Observations

To investigate the hydrodynamic consequences associated with glacier-ice bubbles during melting, two experiments with identical geometries were performed: (1) with clear bubble-free ice (made from freshwater by an ice carving artist), representative of the idealized ice assumed in melt models^36,37^; and (2) with natural glacier ice from Greenland’s Pakitsoq ice margin (containing approximately 200 bubbles cm^−^^3^ and bubble diameter averaging around 0.5 mm)^38,39^. In each case, a vertical ice-wall was immersed in a tank of unstratified saltwater with initial temperature of 4.1 °C and salinity of 28, properties consistent with the springtime ocean conditions at Xeitl Sít’^40^ (see Methods, Supplementary Fig. 1 and Supplementary Video 2 for experimental details). Each experiment was run for 1.5 hours. During that time, particle image velocimetry (PIV) was used to measure the vertically rising near-boundary current adjacent to the ice interface.

**Fig. 2:**
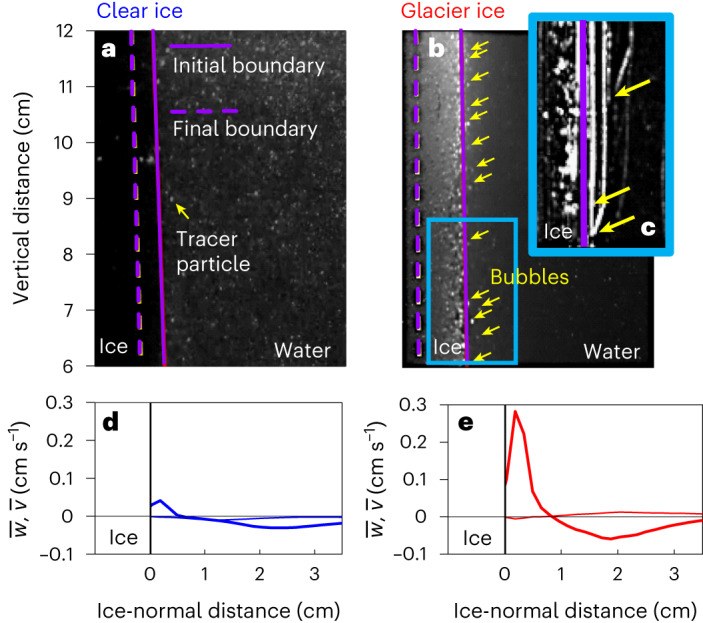
Laboratory observations of melt and hydrodynamics adjacent to clear (bubble-free) and glacier ice during melt. **a**,**b**, Instantaneous hydrodynamic conditions for clear bubble-free ice (**a**) and glacier ice (**b**). Geometry similar to Fig. 1. The solid purple vertical lines show the initial position of the ice face, the purple vertical dashed lines show the final position of the ice face after 1 hour. The clear bubble-free ice in **a** is optically non-reflective because it is completely clear and the small dots in the adjacent water are illuminated tracer particles. The glacier ice in **b** is very reflective because of the bubbles present in the ice as well as in the water. The yellow arrows highlight bubbles that have burst out of the ice and are rising to the tank surface. **c**, A 2-second time-averaged image of the glacier ice case showing the path lines of the bubbles ejecting and moving up the face of the ice as white streaks. The yellow arrows show three instances of actual bubble burst and rise events happening during this 2-second period. **d**,**e**, The measured ice-normal velocity profiles of $\overline{w}$ (vertical velocity component, thick) and $\overline{v}$ (ice-normal velocity component, thin) for the clear (blue, **d**) and glacier (red, **e**) ice, respectively. Source data

In the clear-ice case (Fig. 2a), a thin plume (0.5 cm thick) rises up the face of the ice with a peak velocity of 0.05 cm s^−1^; the maximum velocity is located approximately 0.2 cm from the ice interface (Fig. 2d). From the image sequence, we measure the ice interface melting at a rate of 4 mm h^−1^ and emitting no bubbles.

In the glacier-ice case (Fig. 2b), bubbles of approximately 0.8 mm diameter emerge from the ice interface at an average rate of 4 bubbles cm^−^^2^ min^−1^ and rise upward along with meltwater (Fig. 2c). The ambient melt plume is 0.8 cm thick with a peak average fluid velocity of almost 0.3 cm s^−1^ (Fig. 2e). Bubble bursting events consist of individual bubbles rising at 2 cm s^−1^, an order of magnitude faster than the melt plume. The negative velocity adjacent to the outer edge of the ambient melt plume is a return flow. We find the glacier-ice melt plume is 6 times faster and 2 times wider than the melt plume adjacent to clear ice (compare Fig. 2d,e). The interface melts at 9 mm h^−1^ in the glacier-ice case, 2.25 times greater than the clear-ice case.

## Energetics

To quantify the influence of bubbles on melt-plume dynamics, we compute the average kinetic energy of the mean flow (*KE*) and turbulent flow (*tke*), as well as the dissipation (*ϵ*) of mean and turbulent energy from the experimental PIV measurements within a 6 cm vertical segment of the ice–water interface about halfway up the ice. We find the glacier-ice melt plume has 20 times more *KE* and 6 times more *tke* than the clear-ice melt plume.

The energy source that drives the boundary current flow is buoyancy: for glacier ice, this is supplied by both meltwater and bubble air; for clear bubble-free ice it is supplied by meltwater alone. In the equation governing mechanical energy evolution of buoyant bubble plumes^41^, ‘buoyancy production’ is written as the product of the vertical velocity ($\overline{w}$) and the gravitational anomaly associated with buoyancy (${g}^{{\prime} }$):

1 $$\frac{\partial }{\partial z}\Big[\overline{w}({{KE+tke}})\Big]=\overline{w}{g}^{\,{\prime} }-\epsilon .$$

Here the left-hand side represents the rate of change of energy following a fluid parcel (termed ‘advection’) for a steady flow in which the dominant transport of mechanical energy is in the vertical direction (*z*). The last term on the right-hand side, *ϵ*, is the rate of turbulent and mean energy dissipation through viscosity, which by definition is positive and always acts to remove energy from the flow.

We consider the buoyant production ($\overline{w}{g}^{{\prime} }$) for both glacier- and clear-ice melt, because the buoyant production controls the strength of the boundary-layer flow (*KE*) and energizes its turbulence (*tke*). The buoyant force on a parcel of meltwater is proportional to the density anomaly (Δ*ρ*) of the meltwater (including any ejected air) relative to that of the seawater *ρ*_0_ into which melt is released. This buoyant force scales with the Earth’s gravitational acceleration *g*, ${g}^{{\prime} }=\frac{\Delta \rho }{{\rho }_{0}}g$. The buoyant force for undiluted freshwater immersed in seawater is ${g}^{{\prime} }=0.24$ m s^−^^2^, while for bubbly freshwater (10% air) immersed in seawater, ${g}^{{\prime} }=1.2$ m s^−^^2^, roughly 5 times that of freshwater alone.

**Fig. 3:**
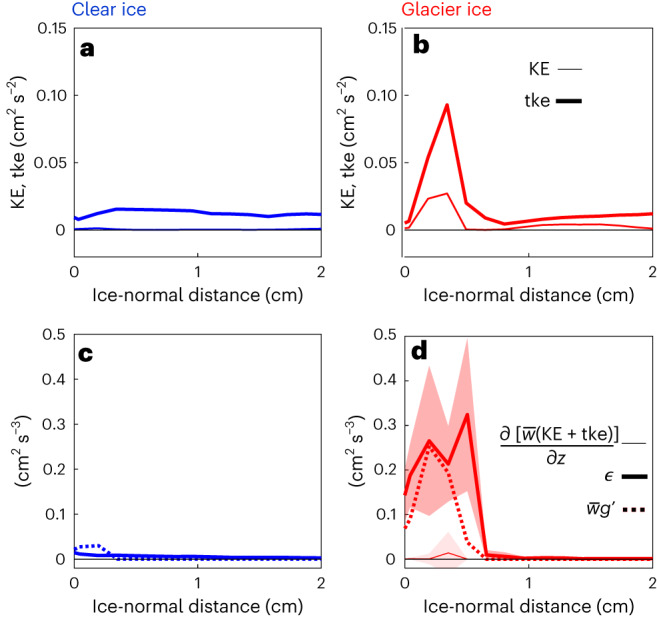
Laboratory observations of the mean *KE* and *tke* adjacent to clear (bubble-free) and glacier ice during melt. **a**,**b**, Vertically averaged *KE* and *tke* for clear ice (blue; **a**) and glacier ice (red; **b**). **c**,**d**, Total energy balance for clear ice (blue; **c**) and glacier ice (red; **d**) showing the energy transport (thin line), dissipation (thick line) and the production due to meltwater and bubble plume rise (dashed line). The thick dashed line is the production term calculated from the plume vertical rise velocity and reduced gravity of the ambient melt plume. The shading around the energy transport and dissipation terms shows the associated standard deviation in measurement of those terms. Source data

Details of the energy balance are shown in Fig. 3, which demonstrates that production and dissipation in the glacier-ice case far exceed that of the clear-ice case (Fig. 3d). Our finding that $\overline{w}{g}^{{\prime} }\approx \epsilon$ in the glacier-ice case implies that local viscous dissipation is nearly balanced by the production of total kinetic energy (*KE* + *tke*) from bubble-induced buoyancy. We note that our imaging system was not of sufficient speed and resolution to capture the bubble explosions themselves (neither the ejection nor their dissipative wake), so these discrete events are omitted from the analysis. In principle, these events probably contribute substantially to energetics very near the ice boundary, and may be responsible for the mismatch in the energy terms near 0.6 cm (Fig. 3). The details of the bubble explosions, how energy flows between *KE* and *tke*, and how this component of the energy is dissipated is beyond the scope of this investigation.

The observed degree of closure suggests that we can estimate the turbulent heat flux (${q}^{{\prime} }$) following a Reynolds analogy (and consistent with the scaling for the transfer coefficient in the three-equation model^36^) in which ${q}^{{\prime} }$ scales with $\sqrt{{tke}}$. As such, the 6 times enhanced *tke* for the glacier ice compared with clear bubble-free ice implies an increase in ${q}^{{\prime} }$ by a factor of 2.4. This estimate of bubble-enhanced heat flux to the ice face is consistent with our observation that glacier ice melts 2.25 times faster than the clear bubble-free ice, because melt rate scales linearly with heat flux.

## Implications at the geophysical scale

While the laboratory experiments allow for isolation of a specific process, they are limited in scale and thus attenuate feedbacks that connect buoyancy input with large-scale convective cells. In addition, our experiments were conducted at atmospheric pressure, so do not include the influence of hydrostatic pressure at depth. Here we explore the implications of our laboratory experiments for melt of a real glacier. We seek to quantify (1) where the additional buoyancy from bubble release has the greatest influence, and (2) how the energy contribution from bubble bursts compares with that of their buoyancy. As an example, we consider Xeitl Sít’ as a prototypical tidewater glacier (terminus scale, seawater properties); because our calculations depend primarily on the properties of glacier ice (that is, bubble size and pressure), they are relevant to an arbitrary submarine glacier face.

**Fig. 4:**
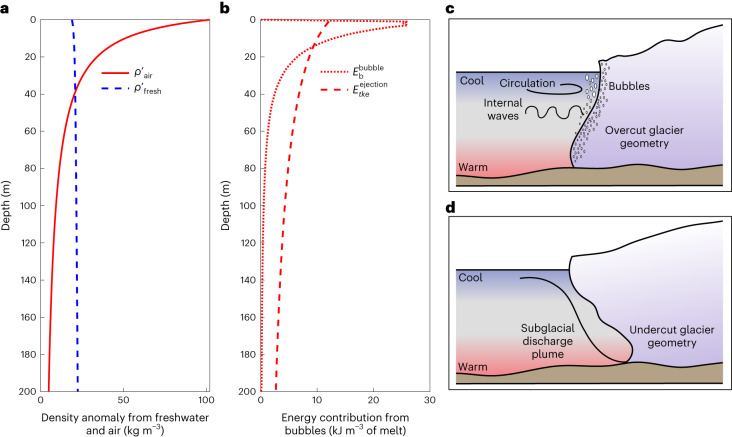
Semi-empirical model results and accompanying schematics showing the influence of bubble ejection on glacier dynamics at field scale. **a**, Buoyant density anomaly associated with freshwater melt (${\rho }_{{{{\rm{fresh}}}}}^{{\prime} }$, blue dashed line) and entrapped air in the glacier ice (${\rho }_{{{{\rm{air}}}}}^{{\prime} }$, red solid line). **b**, Predicted energy contributions from bubble buoyancy (${{E}_{\mathrm{b}}^{\rm{bubble}}}$, red dotted line) and explosive bursts (${E}_{{tke}}^{{{\,{\rm{ejection}}}}}$, red dashed line). **c**,**d**, Schematics consistent with two recent studies of observed terminus shape, overcut (**c**) or undercut (**d**). In **c**, multibeam acoustic imaging finds that some glaciers have overcut sections with melt that increases towards the surface (for example, Xeitl Sít’^43^). We hypothesize that bubble ejection, along with circulation and internal waves could contribute to the overcut geometry, especially outside of the region of influence by the subglacial discharge plume. In **d**, plume-melt theory is used to determine glacier melt and predicts undercut glacier termini, generally biased by the subglacial discharge plume outflow (for example, ref. ^46^). Source data

Plume buoyancy, which is a source of kinetic energy to drive melt, is controlled by the density of the meltwater mixture adjacent to the ice^42^. Previous estimates of the buoyancy input from meltwater assume bubble-free ice, comprising pure freshwater with an approximately constant density anomaly relative to seawater (${\rho }_{{{{\rm{fresh}}}}}^{{\prime} }$) (Fig. 4a, dashed blue line). Meltwater density that accounts for bubbles’ existence (${\rho }_{{{{\rm{air}}}}}^{{\prime} }$) is instead a function of depth. We find the contribution to the density anomaly from bubbles exceeds that of the pure meltwater over the top 40 m (Fig. 4 and Methods).

We estimate the contribution of bubbles to the plume’s *KE* by tracking the buoyant energy of individual bubbles as they rise, expand and ultimately dissolve (${E}_{{\mathrm{b}}}^{{{{\rm{bubble}}}}}$). Below 100 m depths, released bubbles are small and dissolve after rising just 4 m (Supplementary Figs. 4–6). Closer to the ocean surface, released bubbles are larger, rise faster and take more time to dissolve, so can reach the surface intact from as deep as 40 m (dependent on in-ice bubble size and pressure).

We compare the buoyant energy of individual bubbles with the *tke* imparted to the flow through explosive bubble bursts, which is computed as a pressure-work term (${E}_{{tke}}^{{{\,{\rm{ejection}}}}}$). This term depends primarily on the in-ice bubble pressure relative to the seawater hydrostatic pressure; we find it scales more weakly with absolute pressure than the buoyant energy and estimate that ${E}_{{tke}}^{{{\,{\rm{ejection}}}}}$ varies by a factor of 3 over the upper 200 m of the terminus (Fig. 4b). Our analysis suggests that the buoyant energy contribution by bubbles to *KE* at depth is small, but the explosive bubble contribution to *tke* may be important at all depths.

Because of the depth-dependence of bubble energetics, bubbles may not only influence the magnitude of melt, but also the resulting shapes of tidewater glacier termini. The terminus morphology at Xeitl Sít’, for example, can have widespread overcutting^43^, consistent with elevated shallow melt processes (Fig. 4c). High-resolution numerical models^44^ and plume-melt theory (described following refs. ^9,45^), on the other hand, predict glacier termini that are almost universally undercut (Fig. 4d)^46^. Bubbles, along with other sources of near-surface currents^11,13,14^, may contribute to observed variability in terminus shape (Fig. 4c).

## Pathways forward

Explosive bursts of bubbles and their associated buoyancy substantially energize the ocean boundary layer during the melting of natural glacier ice. In the lab, the boundary-layer *KE* and *tke* are increased by a factor of 20 and 6, respectively, for real glacier ice as compared to clear bubble-free ice. This energized boundary layer amplifies the melt rate of glacier ice more than 2.25 times that of clear bubble-free ice. Extending these findings to the geophysical scale suggests bubble dynamics may substantially contribute to submarine ice melt of tidewater glaciers, especially over the upper portion of the submerged glacier termini.

While the acoustic signatures from bubble bursts are a known indicator of ice melt^21^, the hydrodynamic effects of pressurized bubble release have been previously overlooked. Our findings demonstrate bubbles’ corrosive effect on the ice–ocean boundary and their buoyancy contribution to seawater adjacent to the glacier face, both of which may amplify melt over the full glacier terminus. Yet bubble ejection is currently neglected in modelling submarine ice melt at the ice–ocean boundaries of tidewater glaciers, icebergs and ice shelves.

To predict melt of tidewater glaciers, the most commonly used three-equation physics-based model estimates the magnitude of ice melt by seawater^36,37^, coupled with a buoyant plume model that estimates the energy of the buoyancy-fed submarine melt plume^9,45^ (together referred to as plume-melt theory). The three-equation model has been validated with observations beneath sea ice and the horizontal interfaces at the base of ice shelves^36,47,48^, but has limited testing and much discrepancy at vertical glacier-ice interfaces^7,12,11^.

As a pathway forward to incorporate the contribution from bubbles to glacier-ice melt, we suggest a physics-based modification of the standard plume-melt theory. Recent studies have demonstrated that adding realistic horizontal velocities and adjusting the coefficients that control the transfer rates of heat, salt and momentum are ways that plume-melt theory can better match observed melt magnitudes^11,49^. However, instead of ad-hoc adjustments to the coefficients, we suggest that the three-equation model for melt should depend explicitly on properties of the ice. For example, the transfer coefficient should depend on ${E}_{{tke}}^{{{\,{\rm{ejection}}}}}$, which is set by the bubble overburden pressure and size distribution. Similarly, the energy from bubble rise could be explicitly included in the coupled buoyant plume model through the density anomaly ${\rho }_{{{{\rm{air}}}}}^{{\prime} }$. Both could be approximated to scale with depth (as in Fig. 4).

The incorporation of bubble dynamics into the next generation of glacier-ice melt models will increase predicted melt rates at tidewater glaciers, could reconcile inconsistency between observed and predicted termini geometry (undercut versus overcut), and provide a mechanism for sustained melt in winter months. Findings may improve community predictions of ice-sheet evolution and help to constrain sea-level rise projections.

## Methods

### Laboratory experiments

Two experiments, one with glacier ice and one with clear bubble-free ice, were performed. Each experiment was performed for 1.5 hours. During each experiment, 10 independent 1-minute-long samples of the velocity field were collected to aid in statistics and averaging. Experiments were performed in a 120 × 46 × 52.5 cm (length × width × height) glass tank inside a walk-in freezer kept at 4.1 °C (Supplementary Fig. 1). Aquarium salt was added to the tank water to create a salinity of 28 and the mixture allowed to equilibrate with the freezer ambient temperature before testing was initiated. Ambient waters in the laboratory were initially unstratified, so that the vertical extent of the circulation induced by buoyant meltwater rise is set by the tank depth. The top and bottom boundary conditions require vertical velocity $\overline{w}=0$; such that flow accelerates upward along the ice face until it reaches the tank surface where it is directed away from the ice. The only source of fluid motion is associated with ice melt and its ambient buoyant plumes; in the actual fjord-glacier system there are additional contributions to the ambient flow that could drive melt, not captured in our experiments.

The ice test specimens were 32 × 41 cm and mounted inside foam panels that fitted into the small end of a water tank, such that the ice face was adjacent to the saltwater and oriented vertically (Supplementary Fig. 1). Upon starting the test, the ice block was taken from a chest freezer and placed in the walk-in freezer for 1 hour to allow the ice to warm from the negative temperatures in the chest freezer. The ice was then placed into the tank and left there for 2 hours. Over the period of the experiment the water became stratified by the ice melt, and was not mixed. Between experiments the water temperature in the tank was again allowed to equilibrate to the freezer ambient temperature and the salinity brought back to 28 by adding salt.

The tank was instrumented with a RBR Concerto conductivity, temperature and depth (CDT) sensor, and an Edmonds continuous 532 nm green laser sheet imaged with a EdgerTronic high-speed camera viewing the laser sheet from outside the tank. The CTD had a sampling rate of 1 Hz. The camera sampled at 150 Hz with 1,080 × 1,920 pixels and was recorded in 1-minute bursts, 10 times through the experiment. The high-speed camera data were used to perform PIV analysis. Data presented in Fig. 3 are from 1 minute for each of the clear-ice and glacier-ice cases. The camera view field was a 12 cm tall and 5 cm wide window adjacent to the vertical ice face, only the top 6 cm of the field were used for analysis presented in this manuscript. The flow field was seeded with neutrally buoyant glass spheres. Camera images were rectified and calibrated to dimensional information within the illuminated laser field of view using the CalTech Photogrammetry toolbox (https://data.caltech.edu/records/jx9cx-fdh55). The PIV correlation was performed using MatPIV with an interrogation window of 32 × 32 pixels (Supplementary Fig. 2). Both global and peak height filters were applied to the resulting velocity field from the PIV correlations^50^. Mean PIV velocities compared within 5% of an in-situ acoustic Doppler velocimeter.

### Ice characteristics

In this study, Greenland glacier ice from the Pakitsoq ice margin (69° 25.83′ N, 50° 15.20′ W) was used, containing 200 bubbles cm^−^^3^ and in-ice bubble diameter averaging around 0.5 mm^38,39^. These properties are consistent with 10% trapped air by volume when the bubbles close off at local atmospheric pressure^17,51^. Because this ice had been in storage for nearly two decades, bubble pressures are probably lower than in-situ at the time of coring. Samples 15 × 20 cm were frozen within a larger block of bubble-free freshwater ice to form the 32 × 41 cm face.

### Flow and energy analysis

For the glacier ice, rising bubbles were identified in the flow field PIV observations by removing any instantaneous velocities greater than 1 cm s^−1^, which was found to be the lower end of the bubble rise velocity range (1–2.5 cm s^−1^) by tracking individual bubbles in the rectified camera images. As such, all analysis presented in the manuscript considers only the flow field velocities and turbulent quantities for both the clear- and glacier-ice experiments with bubble velocities removed. Additionally, all energy quantities are reported in per unit volume. Results are from 1 minute of collected images. For the analysis, the flow field was decomposed into mean and fluctuating components

2 $$w(\,y,z,t)=\overline{w}(\,y,z)+{w}^{{\prime} }(\,y,z,t),$$

3 $$v(\,y,z,t)=\overline{v}(\,y,z)+{v}^{{\prime} }(\,y,z,t),$$

where $w$ and $v$ are the vertical (*z*) and ice-normal (*y*) velocity vectors varying as a function of time (*t*), respectively, $\overline{w}$ and $\overline{v}$ are the mean velocity vectors, and ${w}^{{\prime} }$ and ${v}^{{\prime} }$ the corresponding fluctuating components. To evaluate terms in the kinetic energy equation (equation (1)) given the two-dimensional (2D) limitations of PIV, we assume $\overline{u}=0$ (cross-ice velocity component) because the flow is nominally 2D, so the mean kinetic energy is ${{KE}}=(1/2)({\overline{v}}^{\,2}+{\overline{w}}^{\,2})$. In contrast, we assume horizontal isotropy in the turbulent components so that ${u}^{{\prime} 2} \approx {v}^{{\prime} 2}$ and the turbulent kinetic energy (*tke*) is

4 $${{tke}}=\frac{1}{2}\left(2\overline{{v}^{{\prime} 2}}+\overline{{w}^{{\prime} 2}}\,\right).$$

The dissipation rate of *tke* (*ϵ*) is traditionally computed from products of velocity gradients and formally has 12 terms, only 5 of which can be computed from 2D observations. By assuming homogeneous and isotropic turbulence, the remaining terms can be approximated from the measured ones, allowing us to calculate it as

5 $$\epsilon =\nu \left(2\overline{{\frac{{\mathrm{d}}{w}^{{\prime} }}{{\mathrm{d}}z}}^{2}}+2\overline{{\frac{{\mathrm{d}}{v}^{{\prime} }}{{\mathrm{d}}y}}^{2}}+3\overline{{\frac{{\mathrm{d}}{v}^{{\prime} }}{{\mathrm{d}}z}}^{2}}+3\overline{{\frac{{\mathrm{d}}{w}^{{\prime} }}{{\mathrm{d}}y}}^{2}}+2\overline{\left(\frac{{\mathrm{d}}{w}^{{\prime} }}{{\mathrm{d}}y}\frac{{\mathrm{d}}{v}^{{\prime} }}{{\mathrm{d}}z}\right)}\right),$$

following ref. ^52^, where *ν* is the kinematic viscosity of the fluid. While the boundary layer is neither homogeneous nor isotropic, this formulation includes both the shear and compressive components of the dominant flow’s strain rate tensor; therefore it is probably a good representation of the dissipation and its structure. However, our measurements resolve only part of the turbulent velocity spectrum, so we apply the methods of ref. ^53^ and fit the Nasmyth universal spectrum to shear spectrum over wavelengths >5 mm. The dissipation is then equal to the integral under the shear spectrum multiplied by 7.5*ν*. Supplementary Fig. 3 shows the ice-parallel wavenumber shear spectra (*S*_d*w*/d*y*_) for the clear-ice and glacier-ice cases with increasing distance from the ice face. The black contours are idealized Nasmyth spectra for the dissipation rates labelled in the figure, the region where the lab data roll off falls away from the idealized Nasmyth spectrum is considered the noise floor for the measurements. We see that the clear and the glacier ice have the same roll off far from the ice face.

In the derivation of equation (1) the divergence theorem was applied assuming a steady, 2D flow so that the material derivative is reduced to the vertical transport term shown. We find the steady state assumption valid because the *tke* changed by only 10% over the course of a 1-minute sampling window and mostly due to the variability in bubble dynamics within our laser sheet window, not due to changes in the mean circulation or the flux of air or meltwaters emerging from the ice.

The reduced gravity term in equation (1), ${g}^{{\prime} }=g({\rho }_{0}-\rho )/{\rho }_{0}$, accounts for added buoyancy due to the ambient melt-plume buoyancy, where *g* is the acceleration due to gravity, *ρ*_0_ is a reference water density far from the ice face and *ρ* is the local water density. Both the clear and the glacier ice have reduced gravity due to the ambient melt plume. For the ambient meltwater, we estimate ${g}^{{\prime} }$ assuming the ambient melt plume has a Δ*ρ* = 1 kg m^−^^3^ applied over the thickness of the ambient melt plume. The buoyant force on a parcel of meltwater is proportional to the density anomaly (Δ*ρ*) of the meltwater (including the ejected air for the glacier-ice case) relative to that of the seawater *ρ*_0_ into which the melt is released. This buoyant force scales with the Earth’s gravitational acceleration *g*, ${g}^{{\prime} }=\frac{\Delta \rho }{{\rho }_{0}}g$. Seawater has a typical density of 1,025 kg m^−^^3^ and freshwater 1,000 kg m^−^^3^, so the buoyant force for undiluted freshwater immersed in seawater is ${g}^{{\prime} }=0.24$ m s^−^^2^. When glacier ice (formed with 10% air) melts at atmospheric pressure, it produces a bubbly meltwater mixture of 90% freshwater (*ρ* = 1,000 kg m^−^^3^) and 10% air (*ρ* = 1.4 kg m^−^^3^). The combined density is 900 kg m^−^^3^, yielding a buoyant force of ${g}^{{\prime} }=1.2$ m s^−^^2^. In reality, meltwaters are diluted by the ambient seawater; the buoyant force on a meltwater mixture is reduced by a factor proportional to its dilution and will also be reduced as bubbles dissolve (Supplementary Fig. 5). The density anomaly is computed for the change in density between fresh and saltwater for the freshwater contribution and between the air-leaven freshwater and seawater for the air contribution (density anomaly calculation described further in Geophysical-scale density anomaly calculations).

### Estimation of heat flux ${q}^{{\prime} }$

The transport of heat in a turbulent flow can be estimated using mixing length theory, whereby the mixing by turbulent eddies of size *ℓ* and characteristic velocity $\tilde{u}$ produce down-gradient transport analogous to an elevated molecular (Fickian) diffusivity. Bubbles increase heat transport by enhancing mixing in two ways: (1) from the turbulent wakes of bubbles moving relative to the flow and turbulence associated with bubbles’ additional buoyancy in the near-boundary flow; and (2) from bubble ejection energetics (Fig. 1b shows a schematic of both mechanisms). We define the turbulent eddy diffusivity for heat, *K*_T_, analogous to the turbulent eddy viscosity for momentum, *ν*_T_, using a Reynolds analogy^54^ such that

6 $${K}_{T} \approx {\nu }_{T} \approx \ell \sqrt{{{tke}}},$$

where *ℓ* is a characteristic length scale (the mixing length) and $\sqrt{{tke}}$ represents the characteristic velocity of the turbulent flow. The kinematic heat flux, ${q}^{{\prime} }$, is

7 $${q}^{{\prime} }={K}_{T}\frac{\partial T}{\partial y},$$

where $\frac{\partial T}{\partial y}$ is the wall normal temperature gradient. If we approximate the temperature gradient across the boundary layer as Δ*T*/*ℓ* and assume that the length scale *ℓ* characterizes both the eddy size and the gradient (not unreasonable perhaps), then the heat flux reduces to ${q}^{{\prime} } \approx \sqrt{{{tke}}}\Delta T$, with Δ*T* being the ice–water temperature difference. Given that Δ*T* is approximately the same for the clear-ice and glacier-ice experiments, the above framework is consistent with ref. ^36^, whereby the transfer coefficient and ${q}^{{\prime} }$ scale with the friction velocity as represented by $\sqrt{{{tke}}}$.

### Geophysical-scale density anomaly calculations

The density anomaly associated with freshwater melt and with air-leaven fluid is estimated with the following three equations:

8 $$\rho {{\prime} }_{{{{\rm{fresh}}}}}={\rho }_{0}-(1-{f}_{{{{\rm{air}}}}}(z)){\rho }_{{{{\rm{fresh}}}}}-{f}_{{{{\rm{air}}}}}(z){\rho }_{0}$$

9 $$\rho {{\prime} }_{{{{\rm{air}}}}}={\rho }_{0}-(1-{f}_{{{{\rm{air}}}}}(z)){\rho }_{0}-{f}_{{{{\rm{air}}}}}(z){\rho }_{{{{\rm{air}}}}}$$

10 $$\rho {{\prime} }_{{{{\rm{total}}}}}={\rho }_{0}-(1-{f}_{{{{\rm{air}}}}}(z)){\rho }_{{{{\rm{fresh}}}}}-{f}_{{{{\rm{air}}}}}(z){\rho }_{{{{\rm{air}}}}}$$

where $\rho {\prime}$ is the density anomaly associated with the descriptive subscript; *ρ*_0_ is the ambient seawater density as observed within Supplementary Fig. 10 of ref. ^11^, varying between 1,021 and 1,022.5 kg m^−^^3^ dependent on depth; *f*_air_(*z*) is the volume fraction of air associated with depth, where the volume fraction of air at atmospheric pressure is 0.1 m^3^ per cubic metre of ice^17^; and *ρ*_fresh_ = 1,000 kg m^−^^3^. The fraction of air per unit volume is calculated using Boyle’s Law by

11 $${f}_{{{{\rm{air}}}}}(z)=\frac{{p}_{{{{{\rm{H}}}}}_{2}{{{{\rm{O}}}}}_{{{{\rm{surface}}}}}}\,{f}_{{{{{\rm{air}}}}}_{{{{\rm{surface}}}}}}}{{p}_{{{{{\rm{H}}}}}_{2}{{{\rm{O}}}}}(z)},$$

where ${p}_{{{{{\rm{H}}}}}_{2}{{{{\rm{O}}}}}_{{{{\rm{surface}}}}}}\,{f}_{{{{{\rm{air}}}}}_{{{{\rm{surface}}}}}}$ is the water pressure and volume fraction of air in bubbly ice at the surface (that is, 10 % air), and ${p}_{{{{{\rm{H}}}}}_{2}{{{\rm{O}}}}}(z)$ is the pressure of the water at depth *z*.

### Geophysical-scale bubble energetics analysis

We estimate the energy contribution from each process shown in Fig. 4 based on typical characteristics of glacier ice (bubble size and bubble pressure^17,51^), ocean conditions (temperature, pressure and gas saturation) and the observed vertical scale of ambient melt plumes at Xeitl Sít’^11^. Each contribution (${E}_{{\mathrm{b}}}^{{{\,{\rm{bubble}}}}}$ and ${E}_{{{tke}}}^{{{\,{\rm{ejection}}}}}$) is computed independently, and quantified per unit volume of ice melt.

The energy associated with buoyant bubble rise is computed by accounting for bubble volumetric change due to pressure (Supplementary Fig. 4) and dissolution (Supplementary Fig. 5), as well as rise velocity (Supplementary Fig. 6) change based on size. Our theoretical analysis is based either on analytical or empirical expressions of bubble and gas physics. Upon release, the bubble will also begin to rise; the rise rate of the bubble depends on the bubble diameter and is calculated based on terminal velocity for small-diameter bubbles that are approximate spheres due to surface-tension effects, causing them to rise in a vertical path. The bubble terminal velocity, *w*_T_, is based on the Stokes solution provided by

12 $${w}_{\mathrm{T}}=\frac{1}{18}g \left\{\right. \! d_{\mathrm{bubble}}^{2}\frac{({\,}{\rho}_{\mathrm{sw}}-{\rho}_{\mathrm{bubble}})}{{\mu}_{\mathrm{sw}}}$$

where *g* is gravity, *d*_bubble_ is the bubble diameter, *ρ*_sw_ and *μ*_sw_ are the density and dynamic viscosity of seawater, respectively, and *ρ*_bubble_ is the density of the air bubble (Supplementary Fig. 6)^55^.

Upon release from the ice, the air bubble will begin to dissolve. The bubble dissolution rate increases with decreasing saturation^56^ and with increasing hydrostatic pressure (depth), but is less dependent on temperature^57^. Supplementary Fig. 5 shows bubble dissolution rates as a function of depth and ocean air saturation formulated based on empirical data from refs. ^56,57^. We assume a bubble dissolution rate curve for water fully saturated with air at atmospheric pressure (solid black line) because observations of glacial meltwater within the subglacial melt plume are considered to be fully saturated with air^58^. Dissolution influences the bubble diameter immediately and continuously as it rises.

Additionally, hydrostatic pressure (*P*) change around the bubble will influence its volume (*V*_bubble_) and therefore diameter as it moves from a deeper depth (*z*_1_) to a shallower depth (*z*_2_), *P*(*z*_1_)*V*_bubble_(*z*_1_) = *P*(*z*_2_)*V*_bubble_(*z*_2_). We account for both the decrease in bubble diameter due to dissolution and the increase in bubble diameter due to buoyant rise within this analysis (Supplementary Fig. 7). The balance between bubble dissolution and bubble growth not only influences bubble persistence and diameter within the seawater, but also bubble terminal velocity. Deeper than approximately 40 m, the typical ejected bubble rarely makes it to the surface before complete dissolution occurs (Supplementary Fig. 7, middle). Shallower than the dissolution threshold depth, ejected bubbles may stay intact until they reach the sea surface (Supplementary Fig. 7). When interpreting the left and middle panels of Supplementary Fig. 7, the *x* axis represents the time since bubble release. As depth increases, the time of bubble persistence changes (for example, a bubble released at 200 m dissolves in approximately 3.5 minutes, while a bubble released at 60 m dissolves in approximately 6 minutes). Bubble in-ice pressure also influences its persistence, bubbles with a higher in-ice pressure will persist longer at every depth than bubbles at lower in-ice pressure.

Finally, we estimate the buoyant energy due to bubble rise and the kinetic energy production due to bubble ejection (Fig. 4). The energy due to buoyant bubble rise was estimated by calculating the potential energy associated with air from the bubbles displacing a volume of the meltwater and rising a δ*H* distance through the water using principles of buoyancy by

13 $${E}_{{{{\rm{b}}}}}^{{{\,{\rm{bubble}}}}}(z)=\mathop{\sum }\limits_{b=1}^{n}{\rho }_{{\mathrm{sw}}}(\,{f}_{{{{\rm{air}}}}}(z))g\Delta H,$$

where *f*_air_(*z*) is the volume fraction of air for the distribution of bubbles at each depth *z* per cubic metre of ice. Δ*H* is the associated discrete rise height for every bubble within *f*_air_(*z*) (Supplementary Fig. 7c). Δ*H* changes with bubble size as it rises and dissolves through the water column. The summation is over all bubbles (*b*) at depth *z*.

The energy due to bubble ejection is estimated by integrating the change in bubble pressure over the bubble volume as it expands after ejection,

14 $${E}_{{{{\rm{tke}}}}}^{{{\,{\rm{ejection}}}}}(z)=\int\nolimits_{{V}_{{\mathrm{o}}}}^{{V}_{{\mathrm{f}}}}{P}_{{{{\rm{bubble}}}}}{\mathrm{d}}{V}_{{{{\rm{bubble}}}}}$$

where the integrand limits are the volume of the bubble at ejection, *V*_o_ (Supplementary Fig. 4, solid line), and the final volume of the bubble, *V*_f_ (Supplementary Fig. 4, dashed line), at the point of equilibrium with ambient water pressure. *P*_bubble_ is the bubble pressure and the integral is performed for all bubbles within a cubic metre of ice.

## Online content

Any methods, additional references, Nature Portfolio reporting summaries, source data, extended data, supplementary information, acknowledgements, peer review information; details of author contributions and competing interests; and statements of data and code availability are available at 10.1038/s41561-023-01262-8.

### Supplementary information

Supplementary Information Supplementary history of ice melt experiments, description of Supplementary Audio and Video files, and Supplementary Figs. 1–7.

Supplementary Video 1 Supplementary Video 1 shows a natural light video of a section of an Antarctic ice core from Taylor Glacier (from near surface in ablation area) melting in freshwater (courtesy of Peter Neff and the National Ice Core Laboratory).

Supplementary Video 2 Supplementary Video 2 shows footage taken during the presented experiments of the boundary layer next to the clear ice and the glacier ice.

Supplementary Audio 1 Supplementary Audio 1 features sound collected with a hydrophone from an actively melting glacier underwater.

### Source data

Source Data Fig. 2 Processed data used to create Fig. 2 from main text.

Source Data Fig. 3 Processed data used to create Fig. 3 from main text.

Source Data Fig. 4 Processed data used to create Fig. 4 from main text.

## Data Availability

Processed data are available through the Arctic Data Center at https://arcticdata.io/catalog with 10.18739/A2KK94D6N. Source data are provided with this paper.

## Code availability

Code is available through the Arctic Data Center at https://arcticdata.io/catalog with 10.18739/A2KK94D6N.

## References

[1] Edwards TL (2021). Projected land ice contributions to twenty-first-century sea level rise. Nature.

[2] Oppenheimer, M. et al. Sea level rise and implications for low-lying islands, coasts and communities. In *IPCC Special Report on the Ocean and Cryosphere in a Changing Climate* (eds Pörtner, H.-O., Roberts et al.) (Cambridge Univ. Press, 2019).

[3] Catania G, Stearns L, Moon T, Enderlin E, Jackson R (2020). Future evolution of Greenland’s marine-terminating outlet glaciers. J. Geophys. Res. Earth Surf..

[4] Aschwanden A, Brinkerhoff D (2022). Calibrated mass loss predictions for the Greenland Ice Sheet. Geophys. Res. Lett..

[5] Straneo F (2019). The case for a sustained Greenland Ice Sheet-Ocean Observing System (GRIOOS). Front. Mar. Sci..

[6] Hock R, Hutchings JK, Lehning M (2017). Grand challenges in cryospheric sciences: toward better predictability of glaciers, snow and sea ice. Front. Earth Sci..

[7] Straneo F, Cenedese C (2015). The dynamics of Greenland’s glacial fjords and their role in climate. Annu. Rev. Mar. Sci..

[8] Rignot E (2016). Modeling of ocean-induced ice melt rates of five West Greenland glaciers over the past two decades. Geophys. Res. Lett..

[9] Cowton T, Slater D, Sole A, Goldberg D, Nienow P (2015). Modeling the impact of glacial runoff on fjord circulation and submarine melt rate using a new subgrid-scale parameterization for glacial plumes. J. Geophys. Res. Oceans.

[10] Slater DA (2020). Twenty-first century ocean forcing of the Greenland Ice Sheet for modelling of sea level contribution. Cryosphere.

[11] Jackson R (2020). Meltwater intrusions reveal mechanisms for rapid submarine melt at a tidewater glacier. Geophys. Res. Lett..

[12] Sutherland D (2019). Direct observations of submarine melt and subsurface geometry at a tidewater glacier. Science.

[13] Slater D (2018). Localized plumes drive front-wide ocean melting of a Greenlandic tidewater glacier. Geophys. Res. Lett..

[14] Cusack J (2023). Internal gravity waves generated by subglacial discharge: implications for tidewater glacier melt. Geophys. Res. Lett..

[15] Bushuk M, Holland DM, Stanton TP, Stern A, Gray C (2019). Ice scallops: a laboratory investigation of the ice–water interface. J. Fluid Mech..

[16] Martinerie P, Raynaud D, Etheridge DM, Barnola J-M, Mazaudier D (1992). Physical and climatic parameters which influence the air content in polar ice. Earth Planet. Sci. Lett..

[17] Gow AJ (1968). Bubbles and bubble pressures in Antarctic glacier ice. J. Glaciol..

[18] Scholander P, Nutt D (1960). Bubble pressure in Greenland icebergs. J. Glaciol..

[19] Dadic R (2019). Temperature-driven bubble migration as proxy for internal bubble pressures and bubble trapping function in ice cores. J. Geophys. Res. Atmos..

[20] Pettit EC (2015). Unusually loud ambient noise in tidewater glacier fjords: a signal of ice melt. Geophys. Res. Lett..

[21] Pettit EC, Nystuen JA, O’Neel S (2012). Listening to glaciers: passive hydroacoustics near marine-terminating glaciers. Oceanography.

[22] Glowacki O, Moskalik M, Deane GB (2016). The impact of glacier meltwater on the underwater noise field in a glacial bay. J. Geophys. Res. Oceans.

[23] Glowacki O, Deane GB, Moskalik M (2018). The intensity, directionality, and statistics of underwater noise from melting icebergs. Geophys. Res. Lett..

[24] Josberger EG (1980). The effect of bubbles released from a melting ice wall on the melt-driven convection in salt water. J. Phys. Oceanogr..

[25] Smith ND, Ashley GM (1996). A study of brash ice in the proximal marine zone of a sub-polar tidewater glacier. Mar. Geol..

[26] McConnochie CD, Kerr RC (2016). The effect of a salinity gradient on the dissolution of a vertical ice face. J. Fluid Mech..

[27] McConnochie CD, Kerr RC (2016). The turbulent wall plume from a vertically distributed source of buoyancy. J. Fluid Mech..

[28] Cenedese C, Gatto VM (2016). Impact of two plumes’ interaction on submarine melting of tidewater glaciers: a laboratory study. J. Phys. Oceanogr..

[29] Cenedese C, Gatto VM (2016). Impact of a localized source of subglacial discharge on the heat flux and submarine melting of a tidewater glacier: a laboratory study. J. Phys. Oceanogr..

[30] McConnochie CD, Kerr RC (2017). Enhanced ablation of a vertical ice wall due to an external freshwater plume. J. Fluid Mech..

[31] Gayen B, Griffiths RW, Kerr RC (2016). Simulation of convection at a vertical ice face dissolving into saline water. J. Fluid Mech..

[32] McConnochie C, Kerr R (2017). Testing a common ice–ocean parameterization with laboratory experiments. J. Geophys. Res. Oceans.

[33] Risso F (2018). Agitation, mixing, and transfers induced by bubbles. Annu. Rev. Fluid Mech..

[34] Poulain S, Villermaux E, Bourouiba L (2018). Ageing and burst of surface bubbles. J. Fluid Mech..

[35] Flury S, Glud RN, Premke K, McGinnis DF (2015). Effect of sediment gas voids and ebullition on benthic solute exchange. Environ. Sci. Technol..

[36] Holland DM, Jenkins A (1999). Modeling thermodynamic ice–ocean interactions at the base of an ice shelf. J. Phys. Oceanogr..

[37] McPhee MG, Maykut GA, Morison JH (1987). Dynamics and thermodynamics of the ice/upper ocean system in the marginal ice zone of the Greenland Sea. J. Geophys. Res. Oceans.

[38] Reeh N, Oerter H, Thomsen HH (2002). Comparison between Greenland ice-margin and ice-core oxygen-18 records. Ann. Glaciol..

[39] Petrenko VV, Severinghaus JP, Brook EJ, Reeh N, Schaefer H (2006). Gas records from the West Greenland ice margin covering the last glacial termination: a horizontal ice core. Quat. Sci. Rev..

[40] Hager AO (2022). Subglacial discharge reflux and buoyancy forcing drive seasonality in a silled glacial fjord. J. Geophys. Res. Oceans.

[41] Lai CC, Socolofsky SA (2019). The turbulent kinetic energy budget in a bubble plume. J. Fluid Mech..

[42] Magorrian SJ, Wells AJ (2016). Turbulent plumes from a glacier terminus melting in a stratified ocean. J. Geophys. Res. Oceans.

[43] Abib, N. et al. Persistent overcut regions dominate the terminus morphology of a rapidly melting tidewater glacier. *Ann. Glaciol.* 1–12 (2023).

[44] Xu Y, Rignot E, Fenty I, Menemenlis D, Flexas MM (2013). Subaqueous melting of Store Glacier, West Greenland from three-dimensional, high-resolution numerical modeling and ocean observations. Geophys. Res. Lett..

[45] Jenkins A (2011). Convection-driven melting near the grounding lines of ice shelves and tidewater glaciers. J. Phys. Oceanogr..

[46] Wood M (2021). Ocean forcing drives glacier retreat in Greenland. Sci. Adv..

[47] Jenkins A, Nicholls KW, Corr HF (2010). Observation and parameterization of ablation at the base of Ronne Ice Shelf, Antarctica. J. Phys. Oceanogr..

[48] Dinniman MS (2016). Modeling ice shelf/ocean interaction in Antarctica: a review. Oceanography.

[49] Jackson RH (2022). The relationship between submarine melt and subglacial discharge from observations at a tidewater glacier. J. Geophys. Res. Oceans.

[50] Sveen, K. MatPIV v. 1.7 (2015).

[51] Herron, S. L. & Langway, C. Derivation of paleoelevations from total air content of two deep Greenland ice cores. *IAHS Publ.* **170**, 283–295 (1987); https://github.com/rdeits/adaptive-PIV/tree/master/thirdParty/MatPIV1.6.1

[52] Delafosse A, Collignon M-L, Crine M, Toye D (2011). Estimation of the turbulent kinetic energy dissipation rate from 2D-PIV measurements in a vessel stirred by an axial Mixel TTP impeller. Chem. Eng. Sci..

[53] Gregg M (1999). Uncertainties and limitations in measuring *ε* and *χ*_*T*_. J. Atmos. Ocean. Technol..

[54] Tennekes H, Lumley JL (1972). A First Course in Turbulence.

[55] Talaia MA (2007). Terminal velocity of a bubble rise in a liquid column. World Acad. Sci. Eng. Technol..

[56] Detsch RM (1990). Dissolution of 100 to 1000 μm diameter air bubbles in reagent grade water and seawater. J. Geophys. Res. Oceans.

[57] Jeffries Wyman J, Scholander P, Edwards G, Irving L (1952). On the stability of gas bubbles in sea water. J. Mar. Res..

[58] Schlosser P (1986). Helium: a new tracer in Antarctic oceanography. Nature.

