# Supplementary material for: Melting of glacier ice enhanced by bursting air bubbles
Source: Nat Geosci. 2023 Sep 7;16(10):871–6. doi: 10.1038/s41561-023-01262-8 (PMC10555835; doi:10.1038/s41561-023-01262-8)
Supplement: Supplementary file 1 — Supplementary history of ice melt experiments, description of Supplementary Audio and Video files, and Supplementary Figs. 1–7. [file 41561_2023_1262_MOESM1_ESM.pdf]

nature geoscience

Article https://doi.org/10.1038/s41561-023-01262-8

Melting of glacier ice enhanced by bursting air bubbles

In the format provided by the authors and unedited

Contents

1 History of bubbly ice melt experiments 1

2 Supplementary Video and Audio File Descriptions 3

3 Supplementary Figures 4

1 History of bubbly ice melt experiments

In the 1970s, a proposition to solve the world's freshwater crisis by towing icebergs equatorward sparked an extensive set of observational programs on melt rates on vertical ice faces [1]. During this period, a team of scientists suspected the bubbles might influence melt and conducted a set of experiments to investigate the influence of bubbles trapped in glacier ice on melt rates [2]. However, because of the dangers of studying ice melt *in situ* on a glacier or iceberg they performed their laboratory experiments on manufactured ice containing CO_2 bubbles at atmospheric pressure instead of actual glacier ice containing high-pressure air bubbles [2].

Results from these experiments found melt rates of clear ice in saltwater to be within 10% of CO_2 ice and the researcher concluded that the air-filled pores in ice have a negligible effect. Results did show that the CO_2 trapped in the porous ice imparted upward momentum to the water adjacent to the ice compared to the clear ice, which is consistent with bubble plume literature [2–4].

Josberger [2] hypothesized that the melt rates were nearly the same between clear and CO_2 ice (even with the excess momentum due to bubble rise) because the CO_2 ice melted with huge cusps on the ice, leaving the ice very rough. In our glacier ice melt experiments, we did not observe these same cusps to form. Additionally, CO_2 (1.5x heavier than air) and the Josberger [2] experiments only measured 5% CO_2 in the ice, where glacier ice has 10% air, as such there is only 1/2 the amount of gas frozen into the ice. Their CO_2 ice was formed under atmospheric pressure, so the bubbles in the CO_2 ice did not take on any pressure, which is much different than glacier ice, where we estimate the pressure of the bubbles in our experiments to be up to 10 bar. It may be that the cusps on the CO_2 ice formed due to the CO_2 bubbles sticking to the ice due to surface tension; since the bubbles were not pressurized, they will not explode out of the ice, instead just slowly melt out, causing ice-flow feedbacks to ablate the ice around the bubble, causing cusps. So it is entirely possible that the Josberger [2] uncertainties in measuring the melt rate are larger than the increase in melt that might have happened during the CO_2 ice experiments. We found 2.25× melting for glacial ice with 10% air bubbles at pressure; even in perfect conditions, the experimental setup of [2] might have only created 1.1 to 1.2× the melt rate, which is within his uncertainty of measuring melt.

Unfortunately, because the Josberger [2] experiments were not preformed with actual glacier ice, the experiments missed the physics that are important,

and the conclusions have contributed to persistent underestimation of the melt of tidewater glacier termini and icebergs. In reassessing the work, Josberger (personal communication, 2022) agrees that these experiments may not capture the effect of bubbles in real glacier ice. The published work using CO_2 ice has been cited since that time to dismiss the role of bubbles and may have contributed to an under-appreciation of the potential for bubbles to impact ice melt and an overall underestimation of the melt of tidewater glacier termini and icebergs [5, 6]. Since then [7] did a laboratory experiment investigating the influence of ice fracturing and ice density on melt rates of brash icebergs; they used real iceberg ice in their melt experiments found that glacier ice does melt faster than clear ice and that the melt rate depends on the ice density (i.e., bubble content in the ice), but they did not investigate the physics behind the differences in ice melt.

2 Supplementary Video and Audio File Descriptions

- **Movie M1** shows a natural light movie of a section of an Antarctic ice core from Taylor Glacier (from near surface in ablation area) melting in fresh water (courtesy of Peter Neff and the National Ice Core Laboratory).
- **Movie M2** shows footage taken during the presented experiments of the boundary layer next to the clear ice and the glacier ice.
- **Audio A1** features sound collected with a hydrophone from an actively melting glacier underwater.

3 Supplementary Figures

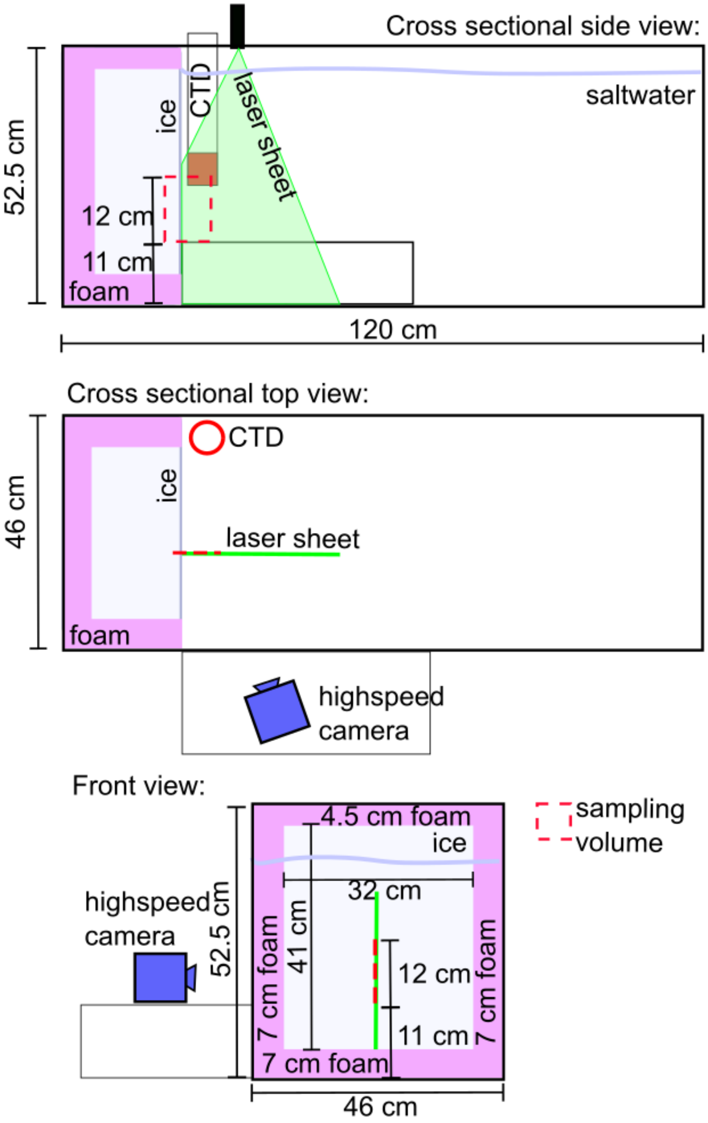

Fig. S1 Experimental setup of the glass tank with insulated ice block at one end. The PIV system was set up as sketched with the perpendicular view of the camera located 50 cm from the tank wall at a 35° angle from the wall plane so that the face of the ice could be captured in each image, when the images were rectified, this angular distortion was removed. The laser sheet was oriented perpendicular to the ice face. The CTD was located 10 cm from the ice face and measured ambient temperature and salinity of the far-field, ambient water. There was a 12 cm field of view of the high speed camera, of which the top 6 cm were used for analysis for this manuscript.

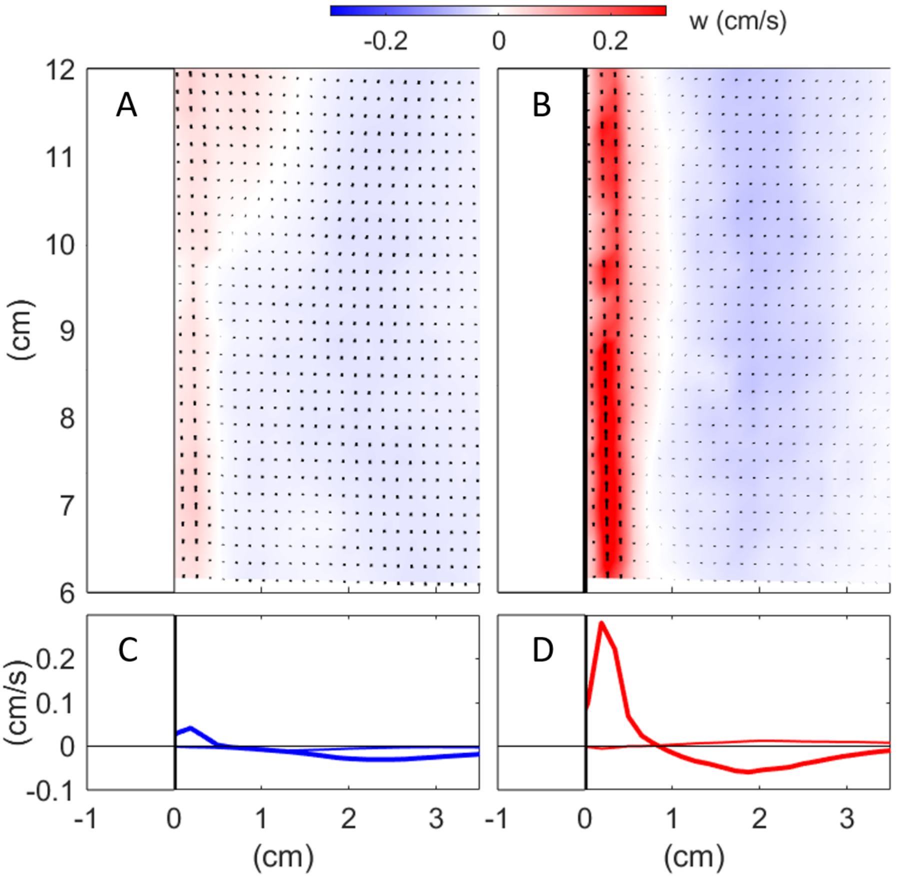

Fig. S2 Average w velocity field (A, B) and mean ice-normal (v, thin) and upward directed (w, thick) velocity profile (C,D) for the clear ice (A, C) and glacier ice (B, D) experiments.

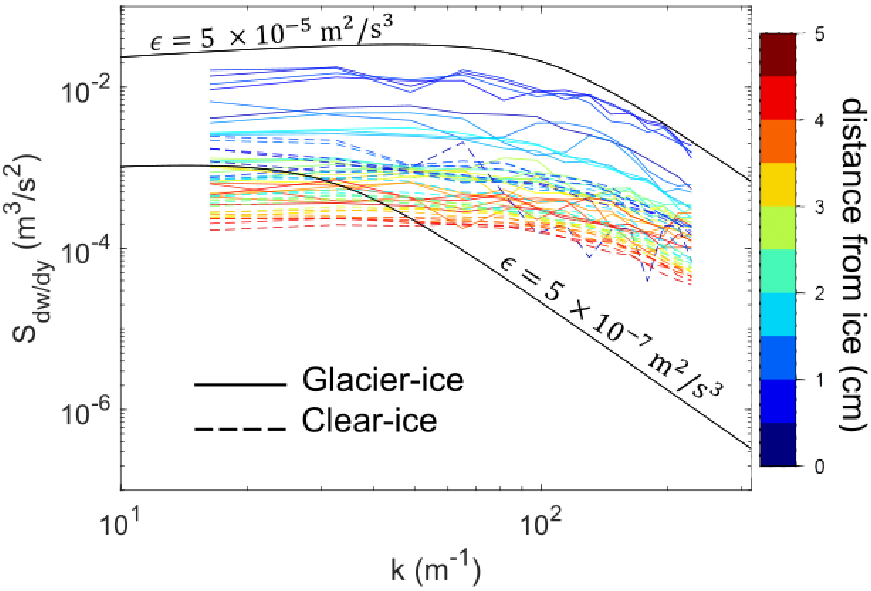

Fig. S3 Wavenumber-shear spectra for the glacier (solid) and the clear (dash) ice measurements. dw/dy spectral estimates were taken at ice parallel contours as shown by color. Black contours show the idealized Nasmyth spectra for the dissipation rates labeled in the figure.

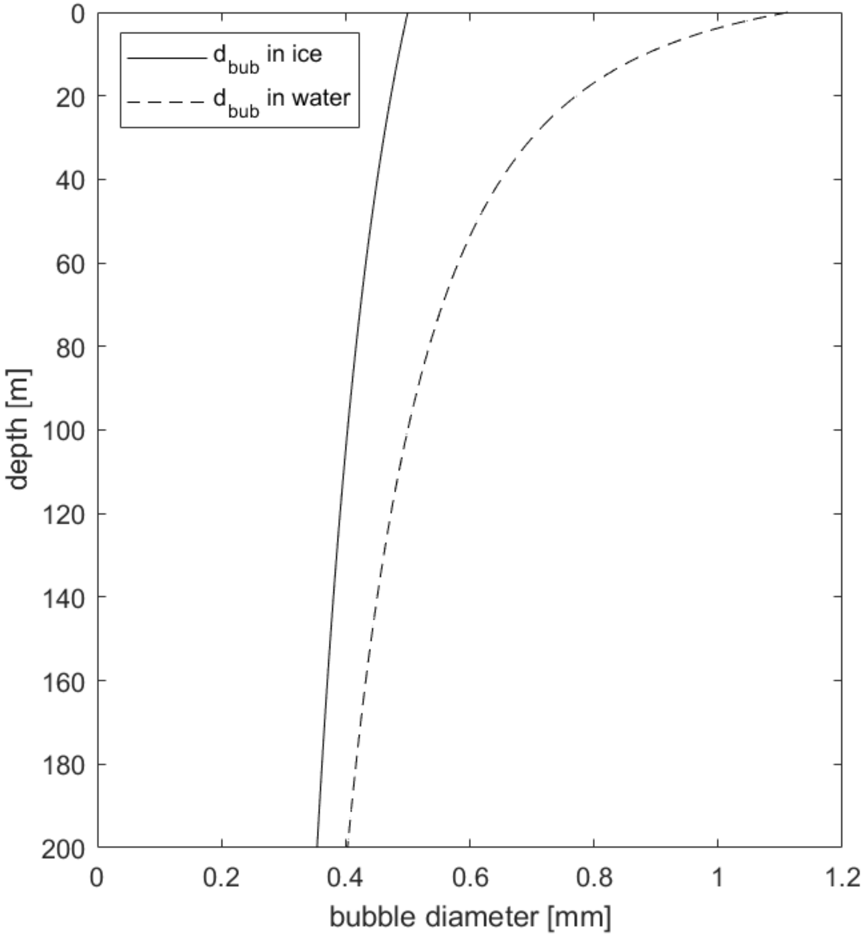

Fig. S4 Bubble diameter in ice and upon release into adjacent seawater as a function of depth at 10 bar of pressure. Initial bubble diameter in ice at surface is 0.5 mm.

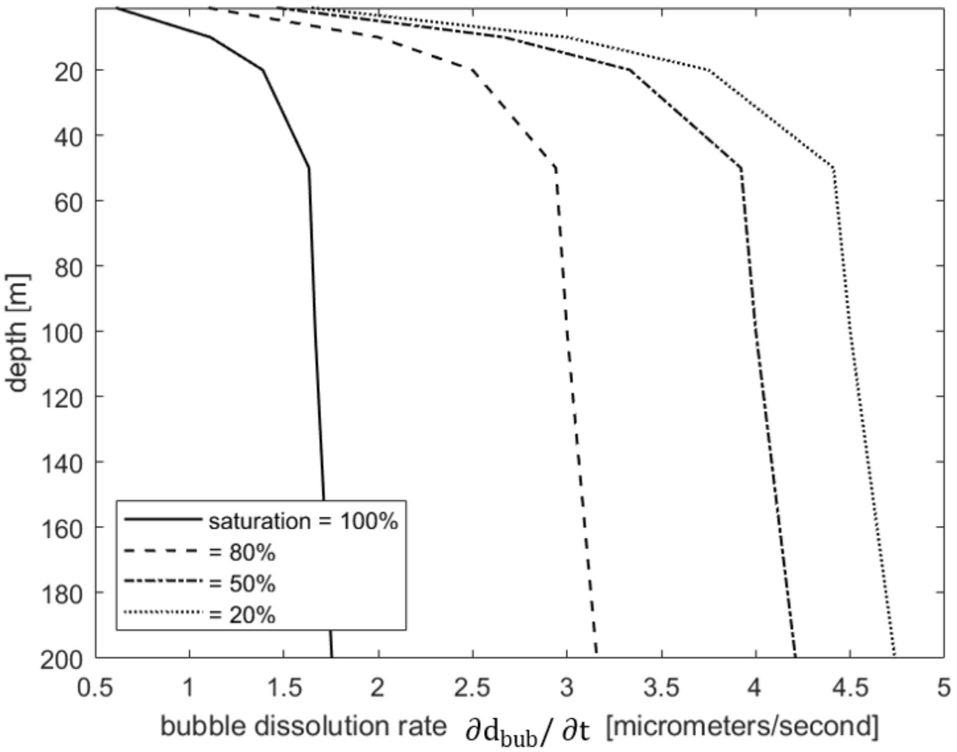

Fig. S5 Bubble dissolution rates with respect to depth and seawater air saturation.

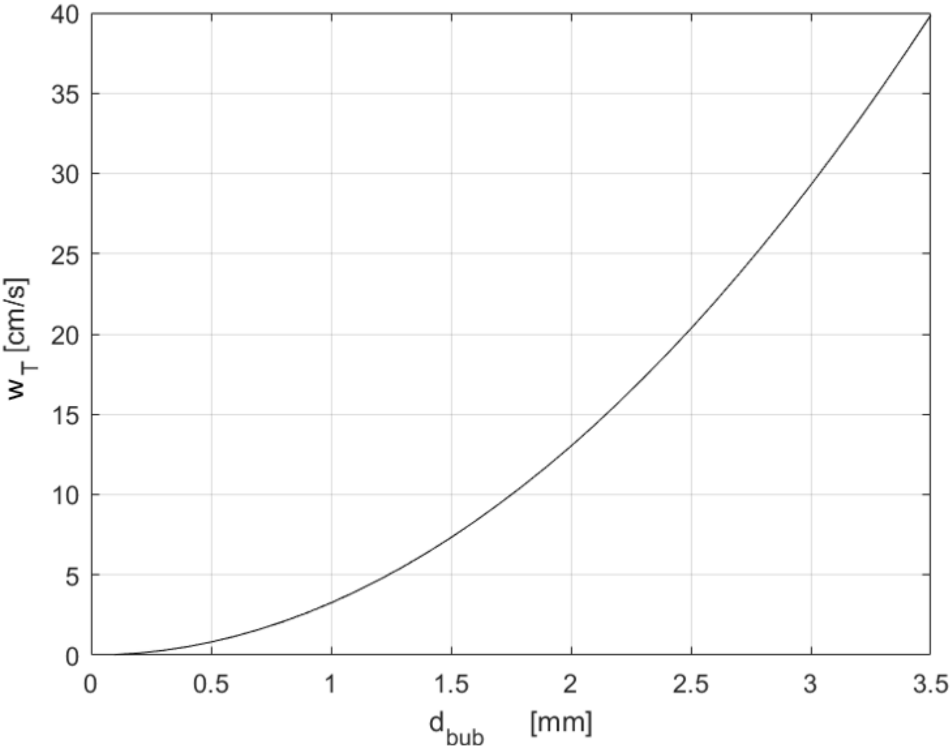

Fig. S6 Empirical terminal velocity, w_T for buoyant bubble rise in a fluid for bubbles of small diameter from Stokes solution.

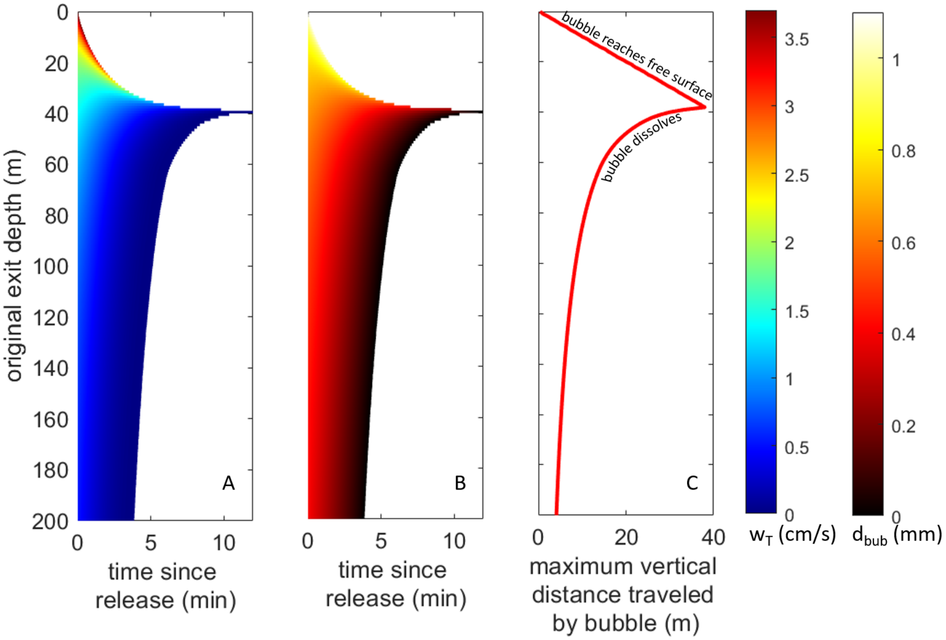

Fig. S7 A) modeled bubble terminal velocity as a function of the original depth where the bubble left the ice and the time since leaving the ice. B) modeled bubble diameter as a function of the original depth where the bubble left the ice and the time since leaving the ice. C) bubble rise distance as a function of the original depth where the bubble left the ice. Figure is made for a bubble pressure of 10 bar and fully air saturated seawater at atmospheric pressure.

References

[1] Husseiny, A.A.: Iceberg Utilization: Proceedings of the First International Conference and Workshops on Iceberg Utilization for Fresh Water Production, Weather Modification and Other Applications Held at Iowa State University, Ames, Iowa, USA, October 2-6, 1977. Elsevier (2013)

[2] Josberger, E.G.: The effect of bubbles released from a melting ice wall on the melt-driven convection in salt water. Journal of Physical Oceanography **10**(3), 474–477 (1980)

[3] Clift, R., Grace, J.R., Weber, M.E.: Bubbles, Drops, and Particles. Dover Publications (2005)

[4] Lai, C.C., Socolofsky, S.A.: The turbulent kinetic energy budget in a bubble plume. Journal of Fluid Mechanics **865**, 993–1041 (2019)

[5] Sutherland, D., Jackson, R.H., Kienholz, C., Amundson, J.M., Dryer, W., Duncan, D., Eidam, E., Motyka, R., Nash, J.: Direct observations of submarine melt and subsurface geometry at a tidewater glacier. Science **365**(6451), 369–374 (2019)

[6] Jackson, R., Nash, J., Kienholz, C., Sutherland, D., Amundson, J., Motyka, R., Winters, D., Skyllingstad, E., Pettit, E.: Meltwater intrusions reveal mechanisms for rapid submarine melt at a tidewater glacier. Geophysical Research Letters **47**(2), 2019–085335 (2020)

[7] Smith, N.D., Ashley, G.M.: A study of brash ice in the proximal marine zone of a sub-polar tidewater glacier. Marine geology **133**(1-2), 75–87 (1996)
